# Temporal Enzymatic Treatment to Enhance the Remodeling of Multiple Cartilage Microtissues into a Structurally Organized Tissue

**DOI:** 10.1002/adhm.202300174

**Published:** 2023-11-12

**Authors:** Ross Burdis, Xavier Barceló Gallostra, Daniel J. Kelly

**Affiliations:** ^1^ Trinity Centre for Biomedical Engineering Trinity Biomedical Sciences Institute Trinity College Dublin Dublin D02 PN40 Ireland; ^2^ Department of Mechanical Manufacturing and Biomedical Engineering School of Engineering Trinity College Dublin Dublin D02 PN40 Ireland; ^3^ Advanced Materials and Bioengineering Research Centre (AMBER) Royal College of Surgeons in Ireland and Trinity College Dublin Dublin D02 PN40 Ireland; ^4^ Department of Anatomy and Regenerative Medicine Royal College of Surgeons in Ireland Dublin D02 YN77 Ireland

**Keywords:** biofabrication, cartilage, chondroitinase‐ABC, ECM remodeling, microtissues, self‐organization, tissue engineering

## Abstract

Scaffold‐free tissue engineering aims to recapitulate key aspects of normal developmental processes to generate biomimetic grafts. Although functional cartilaginous tissues are engineered using such approaches, considerable challenges remain. Herein, the benefits of engineering cartilage via the fusion of multiple cartilage microtissues compared to using (millions of) individual cells to generate a cartilaginous graft are demonstrated. Key advantages include the generation of a richer extracellular matrix, more hyaline‐like cartilage phenotype, and superior shape fidelity. A major drawback of aggregate engineering is that individual microtissues do not completely (re)model and remnants of their initial architectures remain throughout the macrotissue. To address this, a temporal enzymatic (chondroitinase‐ABC) treatment is implemented to accelerate structural (re)modeling and shown to support robust fusion between adjacent microtissues, enhance microtissue (re)modeling, and enable the development of a more biomimetic tissue with a zonally organized collagen network. Additionally, enzymatic treatment is shown to modulate matrix composition, tissue phenotype, and to a lesser extent, tissue mechanics. This work demonstrates that microtissue self‐organization is an effective method for engineering scaled‐up cartilage grafts with a predefined geometry and near‐native levels of matrix accumulation. Importantly, key limitations associated with using biological building blocks can be alleviated by temporal enzymatic treatment during graft development.

## Graphical abstract

Challenges remain in engineering functional cartilage grafts using cellular self‐organization strategies. In this study, it is first demonstrated that microtissue bioassembly generates a more hyaline‐like cartilage tissue compared to a single‐cell scaffold‐free approach. Furthermore, temporal exposure of the developing tissue to chondroitinase‐ABC enhances microtissue fusion and tissue remodeling, which supports the formation of a denser, more mature collagen network that exhibits a biomimetic zonal organization.

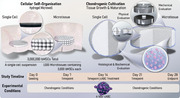

## 1 Introduction

Engineering articular cartilage (AC) that mimics both the structure and composition of the native tissue remains a considerable challenge. Shortcomings associated with traditional top‐down tissue engineering approaches has motivated interest in scaffold‐free strategies that aim to mimic developmental processes to generate a more biomimetic tissue.^[^ ^1^, ^2^, ^3^, ^4^, ^5^, ^6^, ^7^, ^8^ ^]^ These approaches often yield highly biomimetic engineered cartilages as the cells, unconstrained by an interstitial scaffold material, are allowed to interact with and remodel the extracellular matrix (ECM) in a fashion that recapitulates key events in the native tissue's developmental programme.^[^ ^9^, ^10^, ^11^ ^]^ In an attempt to scale‐up the engineering of more complex tissues and organs, emerging scaffold‐free strategies seek to use cellular spheroids, microtissues, and/or organoids as biological building blocks that can be combined to fabricate larger regenerative implants. Such approaches have been applied to multiple tissues, including bone,^[^ ^12^, ^13^, ^14^, ^15^, ^16^ ^]^ cartilage,^[^ ^17^, ^18^ ^]^ vasculature,^[^ ^14^, ^19^, ^20^, ^21^, ^22^, ^23^ ^]^ osteochondral,^[^ ^8^, ^24^, ^25^, ^26^, ^27^, ^28^ ^]^ and liver.^[^ ^29^ ^]^ Compared to more traditional scaffold‐free approaches, that use single cells as the minimal unit building block for generating an engineered tissue, biofabrication using cellular aggregates or microtissues as biological building blocks can offer several benefits.^[^ ^5^ ^]^ For example, biofabrication using multiple microtissues that have been individually engineered in controlled conditions, as opposed to the use of a suspension of single cells, can potentially address concerns around inhomogenous tissue development in highly cellular constructs (i.e., core degradation) and facilitate the generation of larger grafts required for clinical use. Moreover, creating tissues/grafts with a predesigned geometry using a suspension of single cells can be challenging. In contrast, aggregate‐based approaches have been successfully employed to create scaled‐up, functional tissues/grafts with user‐defined geometries using both manual and automated biofabrication methods.^[^ ^12^, ^17^, ^30^, ^31^ ^]^ Specifically, in the field of cartilage tissue engineering, we have demonstrated the capacity to manually bioassemble microtissues within a 3D‐printed polymer framework to generate a biphasic osteochondral implant for joint resurfacing,^[^ ^8^ ^]^ while others have leveraged novel biofabrication strategies (including bioprinting) to spatially organize cell spheroids, organoids, microtissues, and tissue strands for the generation of tissues/grafts with defined geometries and architectures.^[^ ^12^, ^14^, ^23^, ^28^, ^30^, ^32^, ^33^, ^34^ ^]^

Despite considerable progress in this field, ensuring complete fusion between multiple biological building blocks (e.g., cellular spheroids/microtissues) and subsequently directing their remodeling into a unified, geometrically defined tissue with biomimetic organization remains a key challenge.^[^ ^5^, ^23^ ^]^ While such biological building blocks generally fuse together, ^[^ ^14^, ^35^, ^36^, ^37^, ^38^, ^39^, ^40^, ^41^ ^]^ persistent tissue architectures tend to form within each spheroid/microtissue and are apparent throughout the engineered microtissue.^[^ ^12^, ^14^, ^25^, ^28^, ^33^ ^]^ In order to engineer functional tissues, these initial architectures must be effectively remodeled to generate a tissue with native structural organization. Therefore, microtissue “fusion” should not simply be viewed as the merging of individual biological building blocks, but also their remodeling into an organized tissue. In the context of cartilage tissue engineering, recapitulation of the physiological stratification found in native AC remains a key challenge, even when using spheroids/microtissues as part of a tissue engineering strategy. Although the use of instructive scaffolds that provide boundary conditions to the developing tissue have gone some of the way in addressing the challenge of generating a stratified AC,^[^ ^8^, ^42^, ^43^, ^44^ ^]^ complete recapitulation of the zonal organization within engineered cartilage tissues that are compositionally similar to the native tissue remains elusive.

In this study, we first sought to demonstrate the benefits of engineering scaled‐up cartilage grafts via the fusion of multiple cartilage microtissues compared to traditional scaffold‐free strategies where the construct is generated through the self‐organization of individual cells. We then sought to address two of the key challenges associated with the biofabrication of zonally stratified articular cartilage using multiple cartilage spheroids or microtissues, namely, 1) ensuring robust fusion between adjacent microtissues, and 2) directing the remodeling of the initial microtissue architectures into zonally stratified tissue mimetic of AC. To this end, we explored the use of chondroitinase‐ABC (cABC) treatment to help enhance fusion and remodeling of multiple cartilage microtissues into zonally stratified tissue by temporally removing ECM components believed to negatively impact collagen network development. Similar catabolic enzymatic regimes have been successfully employed in vitro to enhance the development of self‐assembled cartilage in other scaffold‐free systems,^[^ ^7^, ^45^, ^46^, ^47^ ^]^ as well as in vivo to improve host‐implant integration in joint resurfacing strategies.^[^ ^48^, ^49^, ^50^, ^51^ ^]^ Here, we sought to determine the impact of such enzymatic treatment on tissue development during the self‐organization of multiple cartilage microtissues, with the aim of encouraging matrix (re)modeling and the generation of a cartilage graft with biomimetic composition and structural organization. Our goal was to engineer a cartilage graft that stained homogenously for cartilage‐specific ECM components (i.e., was devoid of a necrotic core), but whose collagen architecture was heterogenous and mimetic of the native tissue.

## 2 Results

### 2.1 Cartilage Microtissues Self‐Organize into a More Hyaline‐Like Cartilage Tissue Compared to Single Cells

**Figure 1:**
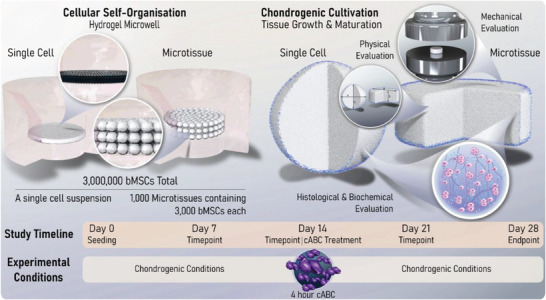
Study Schematic. Two different cellular self‐organization strategies are employed in this study. The first involves the self‐organization of 3 × 10^6^ bMSCs within a nonadherent hydrogel well. The second uses 1 × 10^3^ early‐cartilage microtissues, each containing 3 × 10^3^ MSCs, as biological building blocks. A study timeline is also provided that indicates the weekly endpoints used to map tissue maturation. In one arm of the study, a 4 h chondroitinase‐ABC (cABC) treatment was undertaken on day 14. Finally, a schematic representation of the analytical techniques employed to determine the quality of the self‐organized cartilages generated within this study is provided. Collectively, this work aims to provide a comprehensive timeline for tissue growth and maturation of self‐organized cartilages, as well as determining the effect enzymatic treatment has on the quality of cartilage macrotissues bioassembled using single cells or cartilage microtissues as biological building blocks.

**Figure 2:**
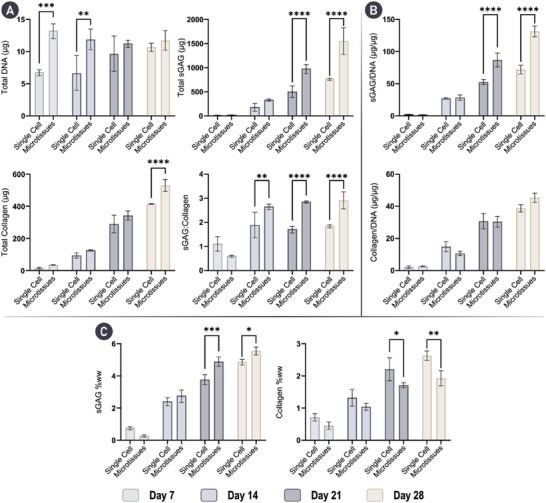
Cartilage microtissues self‐organize a cartilage‐rich ECM. A) Total levels of key cartilage ECM components; DNA, sGAG, and collagen are provided at weekly timepoints for 28 days of chondrogenic cultivation. Total levels of sGAG and collagen are significantly higher in the microtissue group by day 28. Additionally, the sGAG to collagen ratio within the engineered tissues is given. B) sGAG and collagen levels normalized to DNA show that cells within the self‐organizing microtissues produce significantly higher amounts of sGAG at day 21 and 28. C) sGAG and collagen levels normalized to wet‐weight (ww) demonstrate near native levels of sGAG are accumulated in both groups by day 28, with significantly higher levels achieved when using microtissues. However, levels of collagen remain below native levels. *N* = 3, significant differences were determined using an ordinary two‐way ANOVA with a Šídák's multiple comparisons test where, * denote *p* < 0.05, ** denotes *p* < 0.01, *** denotes *p* < 0.001, and **** denotes *p* < 0.0001.

Given there are limited examples directly comparing single cell‐ and microtissue‐based self‐organization strategies, this study first sought to elucidate which method yielded a superior in vitro cartilage. To investigate this, comparable numbers of cells (in either single‐cell or a microtissue format) were placed into hydrogel microwells where neo‐tissue growth and maturation was quantified over 28 days of chondrogenic cultivation, with weekly biochemical and histological evaluation (**Figure** **1**). In both groups, total levels of sulfated glycosaminoglycan content (sGAG) and collagen increased predictably throughout the duration of the culture (**Figure** **2**A). After 28 days, the cartilage engineered via the self‐organization of microtissues contained significantly higher levels of both sGAG and collagen compared to its single‐cell counterpart (twofold higher total sGAG and 1.2‐fold higher total collagen), while the DNA content was comparable in both groups. The sGAG‐to‐collagen ratio within both engineered cartilages revealed a nonphysiological bias toward a GAG‐rich ECM (Figure 2A). Normalization of the absolute amounts of sGAG to DNA quantity demonstrated that biosynthesis of sGAG by resident cells was significantly higher in the microtissue group at days 21 and 28. There were no significant differences in biosynthetic output of collagen per cell (Figure 2B). By day 28, both groups exhibited near‐native levels of sGAG as a percentage of tissue wet weight (4.879 ± 0.174% and 5.546 ± 0.245% for single cell and microtissues, respectively). Despite this evidence of robust chondrogenesis, the levels of collagen (both absolute and normalized) were noticeably lower than those in native AC (Figure 2C).

**Figure 3:**
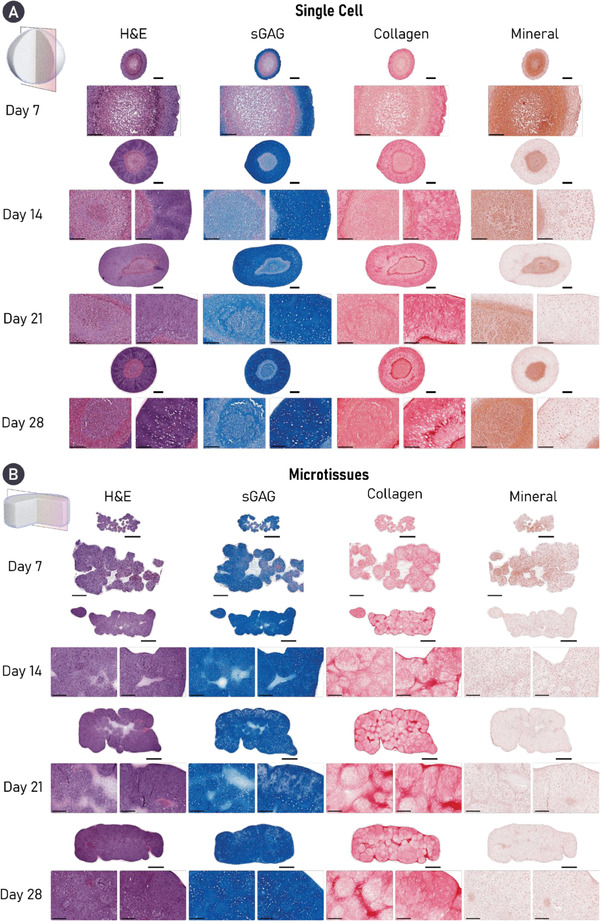
Cartilage microtissues self‐organize a homogenous matrix devoid of mineralization. Histological analysis of engineered cartilage reveals robust accumulation of key cartilage ECM components in both groups, stained for using Alcian blue (sGAG) and picrosirius red (PSR) stain (collagen). Alizarin red staining revealed evidence of cartilage mineralization in tissues engineered using a single‐cell strategy. Additionally, a heterogenous tissue structure and matrix deposition is clearly seen in the single‐cell group, whereas robust microtissue fusion results in homogenous matrix deposition in the microtissue group by day 28. For both histological panels, an overview image is provided as well as zoomed sections for the core (left) and periphery (right) of the construct. A) Scale bars = 500 µm (overview) and 200 µm (zoom). B) Scale bars = 700 µm (overview) and 200 µm (zoom).

While both self‐organization strategies supported a highly chondrogenic phenotype, histological analysis demonstrated clear differences between the two groups. A heterogeneity in cell and matrix phenotype, cellular morphology, and matrix deposition was observed through the depth of the tissues generated in the single‐cell group. In addition, the cell single strategy resulted in a more spherically shaped tissue, while the final shape of the tissues generated using cartilage microtissues better mimicked the shape of the initial hydrogel mold. While the periphery of single‐cell constructs was rich in cartilage matrix components, its core stained weakly for sGAG and was diffusely mineralized. These features were evident as early as day 7 and persisted through the 28 days culture period. Cellular arrangement and morphology also changed through the depth of the single‐cell constructs, appearing hyaline‐like in the construct periphery, but more hypercellular and displaying an aberrant cobblestone morphology in the construct's core (**Figure** **3**A). In contrast, the cartilage engineered via the bioassembly of cartilage microtissues appeared more homogenous. After 14 days, the microtissues had undergone complete fusion, forming a unified macrotissue that stained positively for both sGAG and collagen. Unlike the single‐cell approach, homogenous staining for sGAGs was observed by day 28. Moreover, cells within the microtissue group appeared to be round, and closely resembled native chondrocytes with some native‐like stratification seen in the periphery of the tissue. Importantly, the ECM‐rich cartilage formed via microtissue self‐organization did not mineralize over 28 days of chondrogenic culture, indicative of a more stable hyaline cartilage phenotype (Figure 3B). Collectively, these results provide compelling evidence for the formation of a superior and more homogenous engineered cartilage through the bioassembly of microtissue precursors. Clear evidence of undesirable tissue heterogeneity (i.e., a core deficient in sGAGs) and mineralization was seen in cartilages engineered using single cells as biological building blocks, despite displaying other hallmarks of robust chondrogenesis.

### 2.2 Enzymatic Treatment Supports the Development of a More Biomimetic Cartilage Tissue

**Figure 4:**
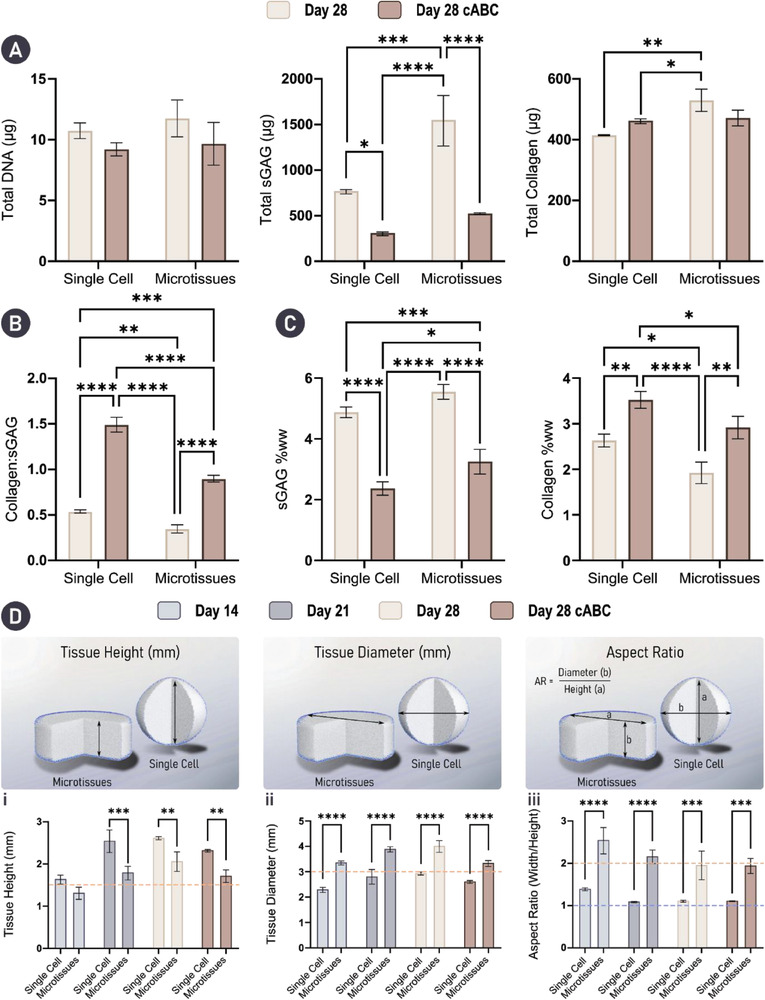
Enzymatic treatment results in a biomimetic ECM composition. Engineering with microtissues results in significantly better shape fidelity. A) Biochemical analysis of the engineered tissues indicated that cABC treatment did not affect total levels of DNA or collagen. However, enzymatic treatment did significantly decrease total levels of sGAG. B) Treatment using cABC significantly improved the collagen to sGAG ratio in both groups. (Caption continued on the following page). C) sGAG and collagen levels as a percentage of tissue wet‐weight (ww) show significant reduction in sGAG content with a concomitant significant increase in collagen content in both groups. Here, sGAG makes up a significantly larger portion of the total tissue wet‐weight (ww) in the microtissue group compared to the single‐cell group following cABC treatment. *N* = 3, significant differences were determined using an ordinary two‐way ANOVA with a Tukey's multiple comparisons test where, * denote *p* < 0.05, ** denotes *p* < 0.01, *** denotes *p* < 0.001, and **** denotes *p* < 0.0001. D) Physical characterization including tissue: i) height, ii) diameter, and iii) aspect ratio indicates that superior control over tissue geometry is achievable by engineering cartilage macrotissues via the assembly of microtissues when compared to a single‐cell approach. Orange dashed lines denote the starting mold dimensions and aspect ratio. Blue dashed line denotes the aspect ratio (AR) of a sphere (AR = 1). *N* = 3, significant differences were determined using an ordinary two‐way ANOVA with a Šídák's multiple comparisons test where, * denote *p* < 0.05, ** denotes *p* < 0.01, *** denotes *p* < 0.001, and **** denotes *p* < 0.0001.

Since the use of similar enzymatic agents/regimes has shown great promise in enhancing the quality of engineered cartilages through modulation of the developing matrix.^[^ ^7^, ^46^, ^47^ ^]^ We next investigated the effect a similar enzymatic regime would have on the self‐organized tissues generated in this study. To this end, we exposed the engineered cartilages to a cABC solution for 4 h at the mid‐point (day 14) of chondrogenic cultivation. Unsurprisingly, biochemical evaluation of the engineered tissues at day 28 indicated that cABC treatment effectively reduced the total sGAG levels 2.48‐fold and 2.96‐fold in the single cell and microtissue groups, respectively (**Figure** **4**A). There was still significantly higher sGAG/DNA in the cABC microtissue group compared to the cABC single‐cell group (Figure S1, Supporting Information). cABC treatment had no effect on total levels of DNA or collagen (Figure 4A,C and Figure S1, Supporting Information). Despite having no effect on collagen synthesis, enzymatic treatment proved an effective method of altering the ratio of sGAG to collagen within the tissue toward a more collagen rich composition, typical of native AC (Figure 4B,C). These findings support the use of cABC treatment as a strategy to significantly increase the relative amount of collagen within engineered cartilage (Figure 4C).

In the context of engineering functional cartilage grafts, ensuring shape fidelity, that is to say the gross morphology of the final tissue closely matches the intended/designed geometry, as well as generating a mechanical competent graft is of great importance. To evaluate the physical properties of our engineered tissues, height, diameter, and aspect ratio measurements were taken throughout the study (Figure 4D). Collectively, these results indicated that engineering cartilage grafts using microtissues as biological building‐blocks yielded a final tissue with superior shape fidelity. Specifically, consistent vertical and lateral growth over 28 days of culture was observed, with engineered tissues maintaining the initial imparted cylindrical shape. In contrast, the use of single cells resulted in aggressive tissue contraction between days 14 and 21 (Figure 7D‐i). Ultimately, the single‐cell approach resulted in an engineered cartilage that was spherical, indicated by an aspect ratio of ≈1 on days 21 and 28 (Figure 4D‐iii). Enzymatic treatment decreased the overall size (width and height) of the engineered tissues in both groups but did not significantly impact the overall aspect ratio of the construct.

There was a trend toward increased mechanical properties with time in culture for the microtissue group (note it was not possible to mechanically test the single‐cell constructs as they adopted a spherical shape in culture). Despite the dramatic loss of sGAGs with cABC treatment, there was also a trend toward increases in the Young's and dynamic modulus with enzyme treatment. After 28 days in culture, the compressive properties of the graft approached that of normal AC, with a Young's modulus of 0.266 ± 0.157 MPa and a dynamic modulus of 0.821 ± 0.298 MPa for the enzymatically treated cartilages generated using microtissues (Figure S2, Supporting Information).

### 2.3 Enzymatic Treatment Improves Microtissue Fusion and Remodeling

**Figure 5:**
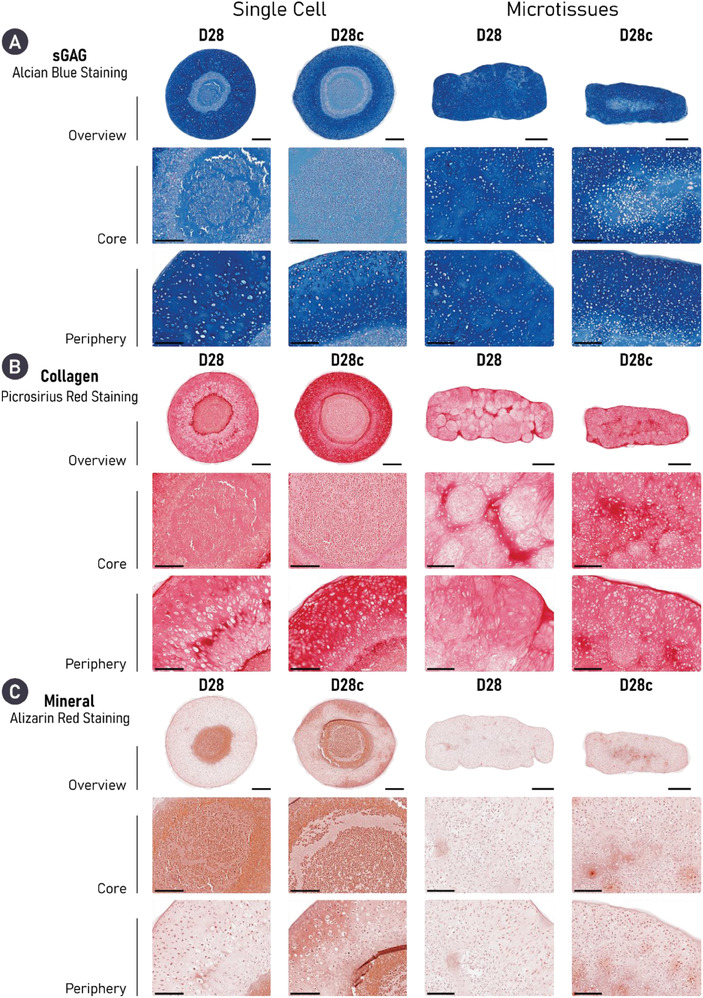
Enzymatic treatment results in densification of the collagen network and notably improves microtissue fusion. Histologically, both engineered cartilages treated with cABC exhibit robust chondrogenesis, as evidenced by diffuse positive staining for sGAG (Alcian Blue) and collagen (PSR stain). cABC treatment causes a denser collagen network in both groups as evidenced by intense PSR staining. Importantly, enzymatic treatment appeared to significantly improve microtissue fusion. However, cABC also appeared to increase mineralization when compared to untreated equivalents. Finally, treatment with cABC results in a smaller, rounder cell morphology in both groups. Scale bars: single cell = 500 µm (overview) and 200 µm (zoom). Microtissue = 700 µm (overview) and 200 µm (zoom).

We next sought to identify, histologically, how cABC treatment influenced matrix structure and composition. Predictably, sGAG staining in both cABC groups appeared weaker compared to the untreated controls, although in line with the biochemical data, the microtissue group appeared to have recovered more sGAG with a less marked decrease in staining intensity (**Figure** **5**A). Despite not significantly increasing total collagen content (biochemically), cABC treatment resulted in a notably more intense collagen staining in both groups indicating a denser network (Figure 5B). As before, in the cABC single‐cell group, a considerable portion of the engineered cartilage was composed of a core, devoid of sGAG and positively stained for mineral deposits (Figure 5C). Consequently, the aforementioned densification of the collagen network was only observed in the periphery of tissue (Figure 5B). It appeared that cABC treatment not only resulted in increased collagen density in the microtissue group, but also facilitated enhanced fusion and remodeling between the microtissue units. As a result of this, there was little to no evidence of their initially spherical geometry (Figure 5). In both groups, cABC treatment appeared to increase mineralization. However, this effect was seen to a greater extent in the single‐cell group, which exhibited diffuse mineral deposition throughout the tissue (Figure 5C). In addition to creating a denser collagen network, cABC treatment also appeared to result in a smaller, rounder cellular morphology in the periphery of the single‐cell group and through the tissue within the microtissue group (Figure 5 and Figure S4, Supporting Information).

### 2.4 cABC Treatment Modulates the Phenotype of Self‐Organized Cartilage

**Figure 6:**
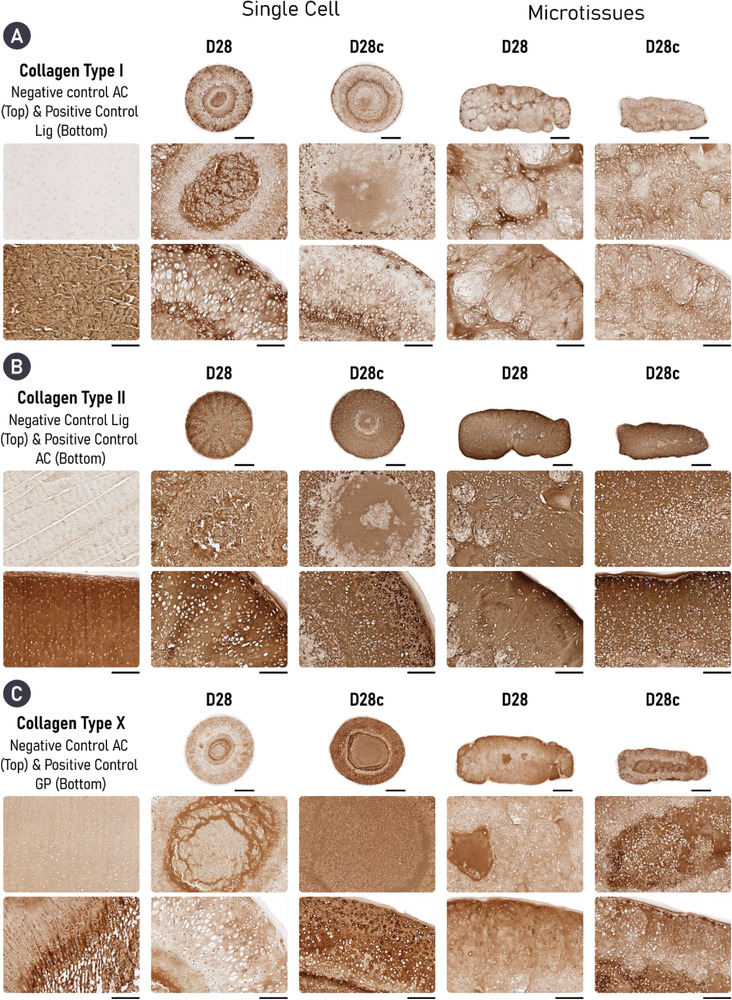
Cartilages engineered treated with cABC exhibit a less fibrocartilaginous, but more hypertrophic phenotype. A) In both self‐organized strategies, cABC appears to reduce the expression of collagen type I. Equally, engineering cartilage via microtissue self‐organization also reduces the expression of collagen type I, with the most pronounced accumulation noted in the D28 single‐cell group. B) All groups diffusely express collagen types II and out of the three collagen sub‐types it is the most abundant. C) Treatment of both engineered cartilages with cABC increases the expression of collagen type X. Scale bar = 700 µm (overview) and 200 µm (zoom).

Having identified changes in ECM composition and structure following enzymatic treatment, we further investigated how removal of sGAGs during early tissue development influences tissue phenotype. To probe cartilage phenotype, immunohistochemical staining for collagen types I, II, and X was undertaken. All samples showed some positive staining for collagen type I, albeit weak, with the greatest expression in the untreated single‐cell group. In both groups, enzymatic treatment decreased the expression of collagen type I, with the most profound effect in the single‐cell group (**Figure** **6**A). All engineered cartilage stained intensely for collagen type II. Relatively homogenous deposition for collagen type II was seen in both microtissue groups, whereas the core of the single‐cell groups expressed less collagen type II than the periphery, an outcome that was exaggerated by cABC treatment (Figure 6B). Collagen type X was least expressed in the untreated single‐cell group, where it was predominantly found in the core of the tissue. In contrast, expression of collagen type X was noted in the periphery of the untreated microtissue group. In both groups, the use of cABC appeared to increase collagen type X deposition (Figure 6C).

### 2.5 cABC Alters Collagen Network Organization and Supports the Development of a Zonally Stratified Tissue

Given the previous histological evidence of changes in the collagen network within the self‐organized cartilage, polarized‐light microscopy (PLM) was employed as a means of investigating how enzymatic treatment influences its spatial organization. Untreated tissues assembled from single‐cell building blocks displayed localized organization. However, as a result of their spherical gross morphology, the overall organization of the collagen network did not closely match that of native AC (Figure S5, Supporting Information). Despite this, cABC treatment resulted in a clear change in the color of the collagen fibers when viewed under polarized light. This color shift from green to orange/red is typically indicative of thickening of the collagen fibers, suggesting that cABC can be an effective method for increasing collagen fiber maturity (Figure S5, Supporting Information).

**Figure 7:**
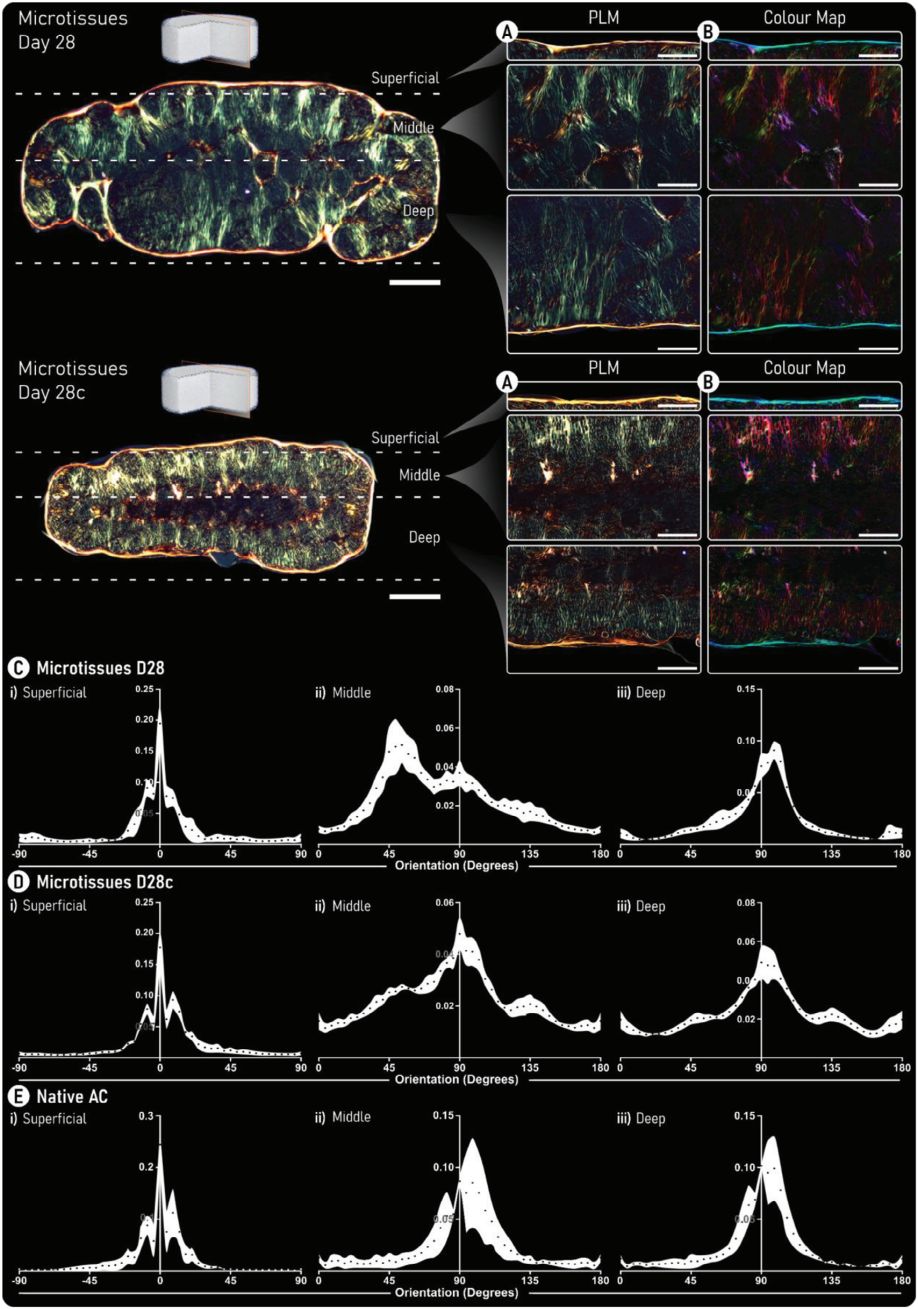
Enzymatic treatment increases collagen fiber thickness and supports the development of a biomimetic collagen network. A) Polarized‐light microscopy (PLM) images of both untreated (top) and enzymatically treated (bottom) tissues generated by self‐organization of cartilage microtissues. B) Color maps generated from PLM images. Here, color hue is used to indicate fiber orientation where, red/pink denotes fibers oriented at 90° and blue/cyan indicates fibers are oriented at 0°. (Scale bars: overview = 500 µm and zoom = 250 µm). C,D) Quantification of the fiber orientation within the i) superficial, ii) middle, and iii) deep zones of the engineered cartilage are provided for C) untreated, D) cABC treated engineered cartilage, and E) native AC. Black data points represent the mean and the white area shows the standard deviation (*n* = 5).

**Figure 8:**
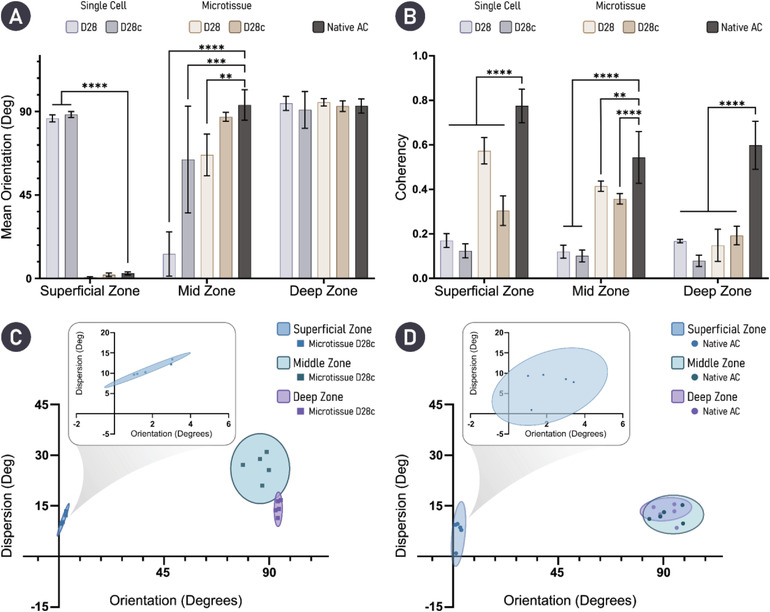
Quantification of collagen fiber orientation indicates that treatment with cABC supports the development of a biomimetic cartilage tissue. A) Mean average fiber orientation within the zones of the engineered tissues is compared with native AC. B) Fiber coherency; values approaching 1 indicate fibers are aligned in the same direction, while a value of 0 indicates dispersion of fibers in all directions. For engineered tissues, *n* = 5 and native tissue *n* = 3. Statistical differences are determined using an ordinary two‐way ANOVA with a Dunnett's multiple comparison test where the experimental groups are compared to the control of native AC. ** denotes *p* < 0.01, *** denotes *p* < 0.001, and **** denotes *p* < 0.0001. C,D) Mean orientation versus dispersion is given for cABC‐treated microtissue engineered cartilage and native AC, respectively.

A similar color shift (from green to yellow/orange) in the collagen fibers could be seen following enzymatic treatment in the microtissue group (**Figure** **7**A). In the context of collagen network organization, cartilages formed through microtissue self‐organization exhibited superior collagen stratification when compared to a single‐cell approach. Quantification of fiber orientation revealed that through enzymatic treatment, a more biomimetic collagen network was generated (Figure 7C–E). Specifically, in both groups (untreated and cABC), the superficial and deep zones of the engineered tissue closely resembled native AC. However, the middle zone of the cABC‐treated microtissue group more closely matched the fiber orientation and distribution seen in native AC. Further quantification of the collagen fiber directionality within the engineered tissues demonstrated significant improvements when using microtissues compared to single cells. The mean fiber orientation in the superficial zone was not statistically different to native AC for the microtissue groups. In the middle zone, only the cABC‐treated microtissue cartilage exhibited a mean fiber orientation that was not significantly different to native AC. In the deep zone of the tissue, all groups, including native AC, had a mean fiber orientation of ≈90° (**Figure** **8**A). Fiber coherency was inferior in all engineered tissues compared to native AC. However, the highest levels of fiber coherency were seen in the tissues engineered using microtissues as biological building blocks (Figure 8B). Collectively, PLM quantification revealed a superior tissue organization via the self‐organization of microtissues compared to a single‐cell approach. Furthermore, enzymatic treatment during the self‐organization of the microtissues yielded a highly biomimetic collagen stratification that closely mimics native AC (Figure 8C,D).

## 3 Discussion

Here, we report a comprehensive comparison between the quality of engineered cartilage tissues formed via the self‐organization of single cells and microtissues. We demonstrated that both formats support robust chondrogenesis, however the cartilage engineered using microtissues as biological building blocks exhibited a significantly richer ECM with higher biosynthetic output at a cellular level. This culminated in a neotissue with near native levels of sGAG accumulation after only 4 weeks of chondrogenic cultivation. Moreover, we demonstrate that the application of a catabolic enzyme (cABC) during tissue development aids in rebalancing the collagen:sGAG ratio within in vitro engineered cartilages, which are typically sGAG rich but collagen poor. Ultimately, cABC treatment promoted maturation of the collagen network within self‐organized cartilage, resulting in the development of a highly biomimetic collagen network with a native‐like zonal stratification.

In this study, we observed that employing a microtissue‐based strategy not only promoted superior cartilage ECM accumulation, but also supported the development of a graft with more spatially homogenous ECM deposition. Specifically, a single‐cell approach supported noticeable heterogeneity within the engineered cartilage after 28 days of chondrogenic cultivation. Similar findings have been reported in the literature, whereby unfavorable radial heterogeneity in cell phenotype, matrix stratification, and occasionally the formation of a necrotic core have occurred following the chondrogenic induction of large, self‐assembled cellular spheroids.^[^ ^52^, ^53^, ^54^, ^55^, ^56^ ^]^ It seems likely that steep radial chemical and nutrient gradients readily develop within high density spheroidal cultures, leading to appropriate cellular differentiation and ECM accumulation at the circumference/periphery, but poor/off‐target outcomes within the core of the implant. This phenomenon has also been observed in scaffold‐based approaches, where so‐called “core necrosis” has impacted the in vivo therapeutic efficacy an engineered implants.^[^ ^57^ ^]^ Latent forms of growth factors such as TGF‐β have been employed in an attempt to alleviate some of the diffusion‐based limitations, by allowing the potent growth factor to penetrate the core of the engineered tissue prior to its activation and action.^[^ ^58^, ^59^ ^]^ Biofabrication using cellular aggregates or microtissues may represent a more effective method of generating tissues of scale, as homogenous cellular differentiation and ECM production can be better controlled within each individual biological building block. Microtissue building blocks have previously been used effectively to engineer millimeter scale tissues for cartilage,^[^ ^17^, ^33^, ^34^, ^60^ ^]^ bone,^[^ ^12^ ^]^ and osteochondral^[^ ^27^, ^28^, ^30^ ^]^ applications without obvious nutrient diffusion limitations. Further work is required to demonstrate that the proposed microtissue approach can be used to engineer more geometrically defined cartilaginous grafts.

Engineering cartilage grafts with numerous microtissues also yielded a tissue with superior shape fidelity when compared to a single‐cell approach. It has been previously demonstrated that controlling tissue development and final geometry is achievable via self‐organization of mesenchymal stem/stromal cell (MSCs) through the use of bioprinting^[^ ^42^, ^61^ ^]^ as well as permanent^[^ ^46^, ^62^, ^63^, ^64^, ^65^ ^]^ or transient cell adhesive substrates.^[^ ^66^ ^]^ In this study, we found that MSCs seeded into a nonadhesive hydrogel well resulted in the formation of a large spherical aggregate, poorly suited as a tissue graft for biological joint resurfacing. This could be a result of increased cell‐mediated contractions in MSC‐only cultures. The use of protein‐coated transwell membranes has previously been shown to allow the formation of cartilage disks rather than spheroids via the self‐organization of single cells.^[^ ^3^, ^46^, ^62^, ^63^, ^64^, ^65^ ^]^ However, the use of such modified membranes restricts the capacity to engineer user‐defined/complex tissue geometries and can make the removal of the engineered tissue/graft difficult. As such, previous successful attempts of self‐assembling single cells into a cylindrical construct in a nonadhesive hydrogel well have employed chondrocytes.^[^ ^10^, ^46^, ^47^ ^]^ The data presented here and in literature suggest that engineering tissues with high shape fidelity via the self‐organization of single cells is challenging and the success, at least in terms of shape fidelity, of an approach is closely coupled to the selected cell type. Our work indicates that using microtissues as biological building blocks can significantly improve the control over final tissue geometry and could provide a relatively simple platform for engineering patient‐specific grafts with user‐defined geometries.

Although microtissue self‐organization enables the engineering of matrix‐rich tissues at a millimeter scale with considerable control over macrotissue morphology, fusion between adjacent microtissues and in particular their (re)modeling into grafts with biomimetic matrix organization remains a challenge. Here, we demonstrate that the temporal introduction of an exogenous remodeling enzyme, chondroitinase‐ABC (cABC), enhances microtissue fusion and macrotissue (re)modeling. Numerous different strategies have previously been employed to enhance the functional development of self‐organized cartilage, including temporal^[^ ^67^ ^]^ and spatiotemporal^[^ ^62^ ^]^ exposure to physiologically relevant growth factors,^[^ ^62^, ^67^ ^]^ physical confinement,^[^ ^68^ ^]^ mechanical stimulation,^[^ ^69^ ^]^ and cABC treatment.^[^ ^7^, ^46^, ^47^ ^]^ In line with our findings, previous studies have observed that the relative amount of collagen within engineered tissue significantly increases following cABC treatment.^[^ ^7^, ^47^ ^]^ This change in matrix composition was correlated with significantly enhanced tensile mechanical properties without compromising compressive properties.^[^ ^7^, ^47^ ^]^ Through cleavage of sGAGs during early tissue development, we were also able to rebalance the collagen:sGAG ratio in the developing matrix toward more physiological levels. Interestingly, while temporal enzyme treatment led to a dramatic reduction in sGAGs from the developing tissue, it did not negatively impact the compressive mechanical properties of the engineered graft. This can potentially be explained by the observed changes to the organization of the engineered tissue, and specifically the development of a more biomimetic, arcade‐like collagen structure which is known to play a key role in determining the mechanical properties of skeletally mature AC.^[^ ^70^ ^]^ More significant improvements in tissue mechanical properties might be expected following multiple cABC treatments and/or extended culture periods to allow complete recovery in tissue sGAG content.^[^ ^46^, ^47^ ^]^ Other approaches that could be adopted in the future include the addition of an exogenous collagen crosslinking agent such as lysyl oxidase (LOX‐2), in conjunction with cABC, to improve collagen fibril maturity and tissue functionality.^[^ ^7^, ^45^ ^]^ Despite identifying significant improvements in ECM quality following enzymatic treatment, there were also subtle changes in tissue phenotype. We noted an increased deposition of collagen type X, a marker of hypertrophy, which may limit the use of such engineered cartilages for biological joint resurfacing. In the context of engineering phenotypically stable hyaline‐like cartilage, we believe the observed hypertrophic drift could potentially be addressed by using a coculture of MSCs and articular chondrocytes^[^ ^71^, ^72^, ^73^ ^]^ and/or the use of a dynamic culture regime,^[^ ^42^, ^74^, ^75^, ^76^ ^]^ both of which have been successfully employed in other studies. Such approaches could enable the generation of scaled‐up, phenotypically stable hyaline‐like cartilages for use in the regeneration of articular cartilage. Additionally, future studies which aim to evaluate the therapeutic efficacy of similar engineered cartilage grafts should include transcriptomic evaluation of the resident stem cell population as a means of robustly determining cell and tissue phenotype prior to implantation. By doing so we aim to better understand the influence of various conditions within multifaceted biofabrication strategies on the composition of the structural collagenous matrix, in particular the expression of collagen types associated with fibro‐, hyaline‐, and hypertrophic‐cartilages.

Temporal enzymatic treatment also resulted in the formation of thicker, more organized collagen fibers. Others have also reported that the addition of exogenous cABC during cartilage development increases collagen fibril density and diameter.^[^ ^7^, ^46^ ^]^ Here, we leverage the color shift in PLM as a qualitative method of determining an increase in fibril thickness/maturity.^[^ ^77^, ^78^ ^]^ Although this approach provided strong visual evidence that changes within the collagen fibril dimeter/maturity had occurred following enzymatic treatment, future studies will aim to leverage alternative techniques such as scanning electron microscopy as a means of quantifying the changes observed in fibril density and thickness. The removal of small regulatory matrix molecules, such as decorin, known to play a role in regulating collagen fibrillogenesis^[^ ^79^, ^80^ ^]^ has been proposed as a mechanism for how cABC treatment enhances collagen maturation.^[^ ^47^ ^]^ Importantly, following cABC treatment, there was limited evidence of boundaries between adjacent microtissues, and it appears that employing this exogenous catabolic enzyme benefitted tissue (re)modeling. The observed structural and organizational changes to the collagen network could help to explain the encouraging trend toward increases in graft Young's and dynamic modulus following temporal enzyme treatment. Although untreated cartilage microtissues were able to fuse together and form a continuous cartilage, evidence of their initial spherical geometry was still apparent after 28 days of culture. While it remains unclear what impact this “more disorganized” ECM will have on implant functionality, PLM displayed clear improvements in the organization of the collagen network within enzymatically treated cartilages which has implications in the broader field of aggregate‐based tissue engineering. The results of our study suggest that temporal enzyme treatment could be applied to a range of different tissue engineering strategies using cellular spheroids, microtissues, tissue strands, or organoids as biological building blocks to fabricate scaled‐up regenerative implants.

## 4 Conclusion

This work has established a robust platform for engineering biomimetic cartilage tissues via cellular self‐organization. Fusion of microtissue building blocks generated a more hyaline‐like cartilage tissue compared to a more traditional scaffold‐free approach using individual cells. In addition, temporal exposure of the developing tissue to a remodeling enzyme (cABC) modulated matrix composition, enhanced microtissue fusion and tissue remodeling, and ultimately supported the formation of a denser, more mature collagen network that exhibited zonal organization analogous to the native tissue. Our findings support the use of temporal enzymatic treatments when tissue engineering using multiple cellular spheroids, microtissues, or organoids as a means of generating more functional grafts.

## 5 Experimental Section

### Bone Marrow MSC Isolation and Expansion

MSCs were isolated from the femoral shaft of 4 month old pigs under sterile conditions. They were expanded in expansion medium (XPAN), composed of high glucose Dulbecco's modified Eagle's medium (hgDMEM) GlutaMAX containing 10% v/v fetal bovine serum, 100 U mL^−1^ penicillin, 100 µg mL^−1^ streptomycin (all Gibco, Biosciences, Dublin, Ireland) and 5 ng mL^−1^ FGF2 (Prospect Bio) under physioxic conditions (37 °C in a humidified atmosphere with 5% CO_2_ and 5% O_2_). After colony formation, MSCs were trypsinized, counted, re‐seeded at a density of 5000 cells cm^−2^, and expanded until the end of passage 2.

### Construct Self‐Organization and Enzyme Treatment

*Microwell fabrication*: The procedure for fabricating the hydrogel microwell platform and the method of generating cellular aggregates and microtissues were described previously.^[^ ^8^, ^13^ ^]^ The same underpinning methodology was employed herein. Briefly, a novel stamp, fabricated using a Form 3 stereolithography printer (Formlabs, MA, USA), was used as the positive mold for the microwell array. The stamps were processed accordingly post‐printing, cleaned, and gas sterilized using ethylene oxide (EtO) prior to use (Anprolene gas sterilization cabinet, Andersen Sterilizers). Nonadherent hydrogel microwells were fabricated under sterile conditions, by patterning a sterile 4% w/v agarose (Sigma Aldrich) solution using the positive molds. To do so, the sterile 3D‐printed stamps were inserted into molten agarose, taking care to avoid the formation of bubbles. Once cooled, the molds were removed from the solidified agarose, leaving an imprint of 401 microwells within each well. All agarose microwells were soaked overnight in XPAN prior to cell seeding.

*Microtissue generation and macrotissue engineering*: Cartilage microtissues were formed using 3 × 10^3^ cells per microtissue in physioxic conditions (37 °C in a humidified atmosphere with 5% CO_2_ and 5% O_2_). Chondrogenesis was initiated by culturing the cells in chondrogenic differentiation medium (CDM) for 2 days. CDM was formulated by supplementing hgDMEM GlutaMAX with 100 U mL^−1^ penicillin, 100 µg mL^−1^ streptomycin (both Gibco), 1.5 mg mL^−1^ bovine serum albumin, 100 µg mL^−1^ sodium pyruvate, 4.7 µg mL^−1^ linoleic acid, 40 µg mL^−1^ L‐proline, 1 × insulin–transferrin–selenium (ITS), 100 × 10^−9^ m dexamethasone, 50 µg mL^−1^ L‐ascorbic acid‐2‐phosphate (all from Sigma), and 10 ng mL^−1^ of human transforming growth factor‐β_3_ (TGF‐β) (Peprotech, UK). After 2 days, microtissues were liberated from the microwells and harvested for future biofabrication steps.

The capacity to form cartilage macrotissues via the spontaneous self‐organization of either cartilage microtissues or single cells was determined by seeding microtissues or a high‐concentration single‐cell suspension into a custom agarose well. The well was created using sterile 2% w/v agarose cast into a 12‐well plate. The central agarose well was 3 mm in diameter and 1.5 mm in depth. The total number of cells seeded into the agarose well in both groups was 3 × 10^6^, meaning 3 × 10^6^ MSCs in a single‐cell suspension of 8 µL, or 1 × 10^3^ microtissues each containing 3 × 10^3^ MSCs in 8 µL. After seeding the wells, plates were centrifuged at 400 × *g* for 5 min to ensure the single‐cells/microtissues were collected at the bottom of the well. Each macrowell was then topped up with 2 mL of chondrogenic medium and returned to the incubator and cultured in physioxic conditions (37 °C in a humidified atmosphere with 5% CO_2_ and 5% O_2_). After 7 days, microtissues were sufficiently fused to allow the removal of the macrotissues from the seeding well. The cartilage tissues were then cultured for the remainder of the study in a 12‐well plate coated with 2% agarose to prevent cellular attachment.

*Chondroitinase‐ABC treatment*: On day 14, prior to enzymatic treatment, constructs were washed three times in hgDMEM. Following which, they were maintained in an enzymatic solution containing 2 U mL^−1^ cABC (Sigma‐Aldrich) and 0.05 m acetate (Trizma Base, Sigma‐Aldrich) activator in hgDMEM for 4 h in physioxic conditions. After the treatment, the engineered tissues were washed again three times with hgDMEM to ensure removal of any residual cABC before the addition of fresh chondrogenic medium and the continuation of chondrogenic cultivation in physioxic conditions for the remaining 14 days.

### Biochemical Evaluation

After retrieval, samples were washed in phosphate‐buffered saline (PBS) and their wet weight recorded immediately. Samples were digested using an enzyme solution, 3.88 U mL^−1^ of papain enzyme in 100 × 10^−3^ m sodium phosphate buffer/5 × 10^−3^ m Na2EDTA/10 × 10^−3^ m L‐cysteine, pH 6.5 (all from Sigma–Aldrich), at 60 °C for 18 h. DNA content was quantified following digestion using Quant‐iT PicoGreen dsDNA Reagent and Kit (Molecular Probes, Biosciences). sGAG was quantified with 1,9‐dimethylene blue (DMMB) at pH 1.5; metachromatic changes of DMMB in the presence of sGAG were determined using the Synergy HT multidetection microplate reader (BioTek Instruments, Inc.) at 530 and 590 nm. 530/590 absorbance ratios were used to generate a standard curve and determine sGAG concentration of unknown samples, chondroitin sulfate was used as standard (Sigma‐Aldrich). Collagen content was determined using a chloramine‐T assay.^[^ ^81^ ^]^ Samples were first hydrolyzed by mixing with 38% HCL (Sigma) and incubating at 110 °C for 18 h. Next, samples were dried, and the sediment was reconstituted in ultra‐pure H_2_O. 2.82% w/v Chloramine T and 0.05% w/v 4‐(dimethylamino) benzaldehyde (both Sigma) were added and hydroxyproline content was quantified using a Synergy HT multidetection microplate reader at a wavelength of 570 nm (BioTek Instruments, Inc.) with *trans*‐4‐hydroxy‐L‐proline (Fluka analytical) standard. Total collagen was calculated using a hydroxyproline to collagen ratio of 1:7.69.^[^ ^81^ ^]^

### Histological and Immunohistochemical Evaluation

*Histological evaluation*: Samples were fixed overnight at 4 °C using a 4% paraformaldehyde (PFA) solution. After fixation, samples were dehydrated in a graded series of ethanol solutions (70–100%), cleared in xylene, and embedded in paraffin wax (all Sigma‐Aldrich). Rehydrated tissue sections (5 µm) were stained with hematoxylin and eosin (H&E), 1% w/v Alcian blue 8GX in 0.1 m hydrochloric acid (HCL) and counter‐stained with 0.1% w/v nuclear fast red, 0.1% w/v PSR, and 1% w/v alizarin red (pH 4.1) (all from Sigma‐Aldrich) to visualize cellular distribution and morphology, sGAG deposition, collagen content, and mineralization, respectively. Stained sections were imaged using an Aperio ScanScope slide scanner and thickness measurements obtained using Aperio Imagescope.

*Immunohistochemical evaluation*: Prior to staining, tissue sections (5 µm) were rehydrated. For collagen type I and type II staining, antigen retrieval was carried out using pronase (3.5 U mL^−1^; Merck) at 37 °C for 25 min, followed by hyaluronidase (4000 units mL^−1^; Sigma‐Aldrich) at 37 °C for 25 min. For antigen retrieval of collagen type X, pronase (35 U mL^−1^; Merck) was used at 37 °C for 5 min, followed by chondroitinase ABC (0.25 U mL^−1^; Sigma‐Aldrich) at 37 °C for 45 min. Nonspecific sites were blocked using a 10% goat serum and 1% bovine serum albumin blocking buffer for 1 h at room temperature. Collagen type I (1:400; ab138492; Abcam), type II (1:400; sc52658; Santa Cruz), and type X (1:300; ab49945; Abcam) primary antibodies were incubated with tissue sections overnight at 4 °C, followed by the addition of a 3% hydrogen peroxide solution (Sigma‐Aldrich) for 20 min. Next, secondary antibodies for collagen type I (1:250; ab6720; Abcam), type II (1:300; B7151; Sigma‐Aldrich), and type X (1:500, ab97228; Abcam) were incubated for 4 h at room temperature. Finally, samples were incubated for 45 min with VECTASTAIN Elite ABC before staining with ImmPACT DAB EqV (both from Vector Labs).

### PLM and Collagen Alignment Quantification

Sections stained with PSR were imaged using PLM to visualize collagen fiber orientation. Quantification of mean fiber orientation, fiber dispersion, fiber coherency, and the generation of color maps was carried out using previously established methods utilizing the “directionality” feature in ImageJ software as well as the OrientationJ plugin.^[^ ^8^, ^42^, ^43^, ^82^ ^]^ The zones of the engineered tissue were defined as follows: the deep‐zone was characterized as the lower 50% of the tissue, the middle‐zone was the intermediate 40%, and the superficial zone was the top ≈10% of the engineered tissue. Significant tissue contraction in the single‐cell group resulted in the formation of a spherical construct and made selecting suitable regions of interest for directionality quantification challenging. To capture meaningful data from the spherical cross‐sections, the size of the “superficial zone” of the cartilage engineered was increased using single cells. In an attempt to present a complete data set, without bias from this experimental caveat, the single‐cell PLM and complementary quantification were positioned in the Supporting Information. The more regular cylindrical cross‐sectional profile of the microtissue constructs enabled evaluation as intended, by dividing the engineered tissue into zones representative of normal articular cartilage. Multiple sections were taken throughout the engineered samples, from which five were selected for quantification. Data points presented graphically represented the quantification of the fiber directionality from the defined zones of the engineered and native tissue. Orientation graphs showed mean average and standard deviation from the histograms generated using the directionality feature in ImageJ software. 95% confidence ellipses presented in dispersion versus orientation plots were determined using the Real statistics resource pack add‐in for excel.

### Mechanical Evaluation

To investigate how tissue maturation and enzymatic treatment influence the mechanical properties of cartilages engineered via the self‐organization of microtissues, unconfined compressions tests were carried out in a PBS bath using a single column Zwick testing machine (Zwick, Roell, Herefordshire, UK) equipped with a 10 N load cell. To ensure contact between the surface of the constructs and the top compression platen, a preload of 0.05 N was used. A peak of 10% strain was applied at a rate of 1 mm min^−1^ and the equilibrium stress was obtained after a relaxation time of 15 min. After the relaxation phase, five compression cycles at 1% strain and frequency of 1 Hz were applied. The ramp modulus, equilibrium modulus, and dynamic modulus were calculated from the resulting stress/strain curves using established methods.^[^ ^83^ ^]^

### Statistical Analysis

Statistical analysis was performed using GraphPad Prism software (GraphPad Software, CA, USA). The data within this manuscript did not undergo any transformation, was assumed to be normally distributed, and no outliers were removed. Numerical and graphical results were presented as mean ± standard deviation (SD) throughout, and a minimum sample size of 3 (*n* = 3) was used for each statistical analysis. To evaluate differences between two groups at multiple timepoints, an ordinary two‐way analysis of variance (ANOVA) was performed with an appropriate correction/post hoc test for multiple comparisons. The statistical analysis performed in each case is described in the table/figure caption. For all comparisons, the level of significance was determined as follows; ns (not significant) *p* > 0.05, * *p* < 0.05, ** *p* < 0.01, *** *p* < 0.001, and **** *p* < 0.0001.

### Ethics Approval Statement

Not applicable. The cells and biological tissues used within this study were sourced from surplus material from the food industry. The source of this animal cells/tissue was considered excess material from livestock animals that were slaughtered for meat consumption at a local abattoir. As such, no animals were specifically bred or culled for scientific purposes and hence, did not require specific ethical approval.

## Conflict of Interest

The authors declare no conflict of interest.

## Author Contributions

R.B. and D.K. were involved equally in the conception and design of this study. R.B. led and X.B. assisted the experimental work, methodology development, and in data acquisition and analysis. R.B. was responsible for data visualization and prepared the original manuscript draft. R.B., X.B., and D.K. were involved with data interpretation, validation, as well as manuscript review and editing. D.K. provided resources, supervision, project administration, and funding acquisition.

## Supporting information

Supporting Information

## Data Availability

The data that support the findings of this study are available from the corresponding author upon reasonable request.
